# Computer-assisted preoperative planning via digital telemedicine for the treatment of periarticular fractures of the extremities: a multicenter cohort study

**DOI:** 10.1097/JS9.0000000000003502

**Published:** 2025-09-23

**Authors:** Xiaoyang Jia, Kun Zhang, Gengxin Jia, Minfei Qiang, Tianhao Shi, Zhenqi Yang, Qinghui Han, Juzheng Hu, Fengfei Lin, Zhi Yuan, Ying Wu, Yanxi Chen

**Affiliations:** a Department of Orthopedic Surgery, Zhongshan Hospital, Fudan University, Shanghai, China; b Department of Orthopedics, Shanghai Changzheng Hospital, Naval Medical University, Shanghai, China; c Department of Orthopedic Trauma, East Hospital, Tongji University School of Medicine, Shanghai, China; d Department of Trauma Center, Liuzhou Worker’s Hospital, The Fourth Affiliated Hospital of Guangxi Medical University, Guangxi, China; e Department of Orthopaedics, Fuzhou Second General Hospital School of Clinical Medicine, Fujian Medical University, Fuzhou, China; f Department of Orthopedics, Xijing Hospital, Fourth Military Medical University, Xi’a, China; g Department of Biostatistics, Guangdong Provincial Key Laboratory of Tropical Disease Research, School of Public Health, Southern Medical University, Guangdong, Guangzhou, China

**Keywords:** computer-assisted, digital telemedicine, fractures, periarticular fractures, preoperative planning

## Abstract

**Background:**

Accurate preoperative planning is essential for the treatment of complex fractures, but the proportion of patients who have access to this service remains low. Therefore, the purpose was to evaluate the association of computer-assisted preoperative planning via digital telemedicine with the risk of postoperative in-hospital complications for periarticular fractures of the extremities.

**Methods:**

A multicenter retrospective cohort study was performed from January 2010 to December 2019. A total of 11 192 patients (≥18 years) with periarticular fractures of the extremities (proximal humerus, distal humerus, distal radius, intertrochanteric, distal femur, tibial plateau, distal tibia, and trimalleolar) were identified and divided into two cohorts: 7130 (63.7%) patients received conventional preoperative planning and 4062 (36.3%) patients received computer-assisted preoperative planning via telemedicine. Propensity score matching for 23 baseline characteristics yielded 4050 patient pairs. Primary outcome was in-hospital complications (surgical site infection, urinary tract infection, pneumonia, sepsis, thromboembolic event, stroke, and myocardial infarction).

**Results:**

Among 11 192 unadjusted patients, the mean (SD) age was 60.2 (12.3) years, and 7052 (63.0%) were female. After propensity score matching (total 8100 patients, 4050 patients in each group), patients with computer-assisted preoperative planning via telemedicine had the lower in-hospital complications [7.9% (318/4050 patients) vs. 10.9% (442/4050 patients); hazard ratio, 0.72 (95% CI, 0.59–0.89); *P* = 0.002] compared with those with the conventional method.

**Conclusion:**

For patients undergoing complex periarticular fractures of the extremities, the use of computer-assisted preoperative planning via digital telemedicine was associated with a lower incidence of in-hospital complications compared with the use of the conventional method.

**HIGHLIGHTS**

- This study integrated digital telemedicine into the entire process of computer-assisted preoperative planning for periarticular fractures for the first time, achieving remote collaboration among 5 trauma centers and solving the problem of geographical accessibility of high-quality preoperative planning resources.
- Compared to conventional method, computer-assisted preoperative planning via telemedicine was associated with a significantly lower risk of postoperative in-hospital complications for complex periarticular fractures.
- Computer-assisted virtual preoperative planning was efficient, averaging approximately 30 minutes per case within an integrated system, confirming the feasibility and practicality of this remote collaborative model in clinical practice.

## Introduction

Trauma is a major cause of mortality and disability worldwide, and fractures are common sequelae of traumatic injuries^[1,2]^. The incidence of injury-related fractures has been increasing owing to the combined effects of accelerated urbanization, increased motorization, and an aging population^[3–5]^, which has become a major drain on medical resources^[6,7]^. In addition, increasing expectations for the post-fracture quality of life present new challenges for healthcare providers, particularly orthopedists^[8]^. Therefore, ensuring the therapeutic effect of these fractures is imperative, and meticulous preoperative plans may provide an opportunity to achieve this goal. Preoperative planning is widely recognized as the first step, and its importance is summarized by the saying, “failing to plan is planning to fail”^[9,10]^.

Historically, conventional preoperative planning has been performed using hardcopy radiographs and tools such as tracing paper, scissors, and colored pens, or simply mentally by visualizing the procedural steps. However, the conventional methods are neither intuitive nor accurate. The critical details of fractures that have a significant impact on surgery are often overlooked, leading to predictable postoperative outcomes^[10–12]^. In recent years, computer-assisted virtual surgical technology has been increasingly applied to the preoperative planning of fractures to achieve better clinical outcomes^[10–13]^. The primary advantage of computer-assisted preoperative planning lies in its ability to offer surgeons a more accurate and detailed visualization of fracture characteristics. In addition, surgeons can perform virtual surgeries, including simulating fracture reduction and inserting an appropriate internal fixation. However, despite the well-established benefits, few patients in China have access to advanced computer-assisted preoperative planning services. Therefore, a computer-assisted preoperative planning model that is accessible, convenient, and efficient is required to deliver this effective therapy and to address the disparity between current clinical practice and patient needs.

In recent years, the application of digital telemedicine has become increasingly widespread, yielding satisfactory results^[14,15]^. This partially alleviates the limitations of the advanced technology in terms of time and space. However, previous digital telemedicine services have primarily focused on medical activities such as diagnosis, education, and follow-up, and their application and effectiveness in orthopedic preoperative planning remain unclear. Therefore, this study aimed to assess whether computer-assisted virtual preoperative planning via digital telemedicine is associated with a decreased risk of postoperative in-hospital complications and improved functional outcomes among patients with complex periarticular fractures of the extremities. We hypothesized that telemedicine based on virtual surgical planning would result in better outcomes than conventional methods.

## Methods

### Data sources

This retrospective cohort study was conducted using the electronic medical record database of the hospital and follow-up records. The database contains patient demographics at admission, comorbidities, injury details, surgical records, progress notes, and follow-up records, including postoperative clinical outcomes. This research received approval from the Institutional Review Board; however, no informed consent was required as the data were de-identified. This work is in line with the Strengthening of the Reporting of Cohort, Cross-Sectional, and Case-Control Studies in Surgery (STROCSS)^[16]^ and the Standards for Quality Improvement Reporting Excellence (SQUIRE 2.0) criteria^[17]^.

### Study population

**Figure 1.**
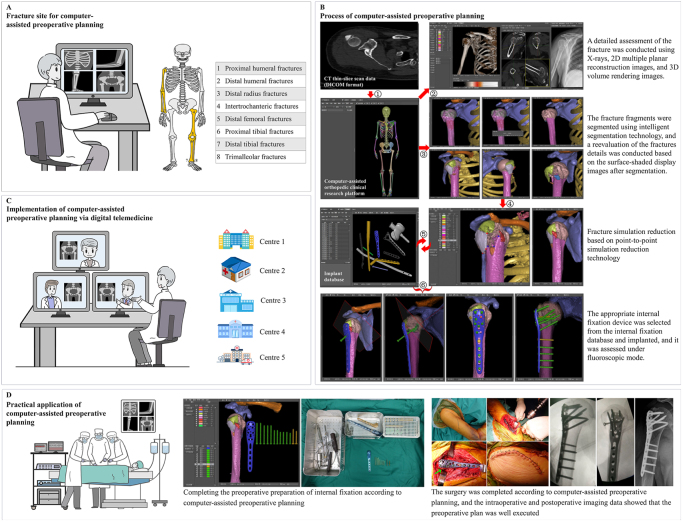
Computer-assisted virtual preoperative planning via digital telemedicine. (A) Computer-assisted virtual preoperative planning was applied to periarticular fractures of the extremities, including proximal humerus, distal humerus, distal radius, proximal femur, distal femur, proximal tibia, distal tibia, and malleolar segment. (B) Flow diagram of preoperative planning with computer-assisted virtual technology. (C) Computer-assisted preoperative planning was used in five trauma centers via digital telemedicine. (D) Practical application of computer-assisted preoperative planning in clinical work.

Between 1 January 2010 and 31 December 2019, all patients aged 18 years and older with periarticular fractures of the extremities (including the proximal humerus, distal humerus, distal radius, proximal femur, distal femur, proximal tibia, distal tibia, and malleolar segment) (Fig. 1A) who underwent surgery less than 3 weeks after the fracture were identified and included. Those fractures, using AO Foundation/Orthopedic Trauma Associated (OTA) fracture classification, included the articular or four-part fracture of the proximal humerus (AO/OTA 11-C), complete articular fracture of the distal humerus (AO/OTA 13-C), complete articular fracture of the distal radius (AO/OTA 2R3-C), trochanteric region fracture of the proximal femur (AO/OTA 31-A, also known as intertrochanteric fractures), complete articular fracture of the distal femur (AO/OTA 33-C), complete articular fracture of the proximal tibia (AO/OTA 41-C, also known as tibial plateau fractures), complete articular fracture of the distal tibia (AO/OTA 43-C), and transsyndesmotic fibula fracture (AO/OTA 44-B, also known as trimalleolar fracture) (Supplemental Digital Content, eMethod One, available at: http://links.lww.com/JS9/F218)^[18]^. Patients who had other types of fractures in the above sites (proximal humerus, AO/OTA 11-A or 11-B; distal humerus, AO/OTA 13-A or 13-B; distal radius; AO/OTA 2R3-A or 2R3-B; proximal femur, AO/OTA 31-B or 31-C; distal femur, AO/OTA 33-A or 33-B; proximal tibia, AO/OTA 41-A or 41-B; distal tibia, AO/OTA 43-A or 43-B; and malleolar segment, AO/OTA 44-A or 44-C) (Supplemental Digital Content, eMethod One, available at: http://links.lww.com/JS9/F218), multiple trauma, open fractures, pathological fractures, previous fractures or surgery at the current fracture site, cognitive impairment preventing them from rehabilitation, opposition to medical advice, treatment with joint replacement, or treatment with external fixator were excluded. Two experienced orthopedic surgeons reviewed the clinical and imaging data (plain radiographs and CT scans) to confirm the fracture classification and patient eligibility.

### Exposure variable

In this study, patients were categorized into two groups based on the type of preoperative planning: the conventional group and the computer-assisted digital telemedicine group. The patients were from five level-1 trauma centers across the country. Conventional preoperative planning was performed using tools such as routine imaging examinations (including hard copies or digital images) and/or simply visualizing the anticipated surgical procedures mentally. For the detailed steps of computer-assisted preoperative planning (Fig. 1B) (Supplemental Digital Content eFig. S1, available at: http://links.lww.com/JS9/F218), thin-slice CT scans from 16- or 32-detector spiral CT scanners (GE LightSpeed; GE Medical Systems) were imported into a computer-assisted orthopedic clinical research platform (SuperImage system, orthopedic edition 1.1; Cybermed)^[10–12]^. A three-dimensional image of the fractured adjacent joint was reconstructed using a surface-shaded display algorithm (Supplemental Digital Content, eFig. S1A, available at: http://links.lww.com/JS9/F218). Fragments can be color-coded for distinction, and any segment can be hidden to facilitate precise evaluation of fracture details. Therefore, unrelated bones could be hidden, allowing for better visualization of articular fragments (Supplement Digital Content, eFig. S1B, C, available at: http://links.lww.com/JS9/F218). The reduction process was simulated, and the appropriate number and size of internal fixations were selected and virtually implanted (Supplement Digital Content, eFig. S1D–F, available at: http://links.lww.com/JS9/F218). Consequently, the following aspects were assessed and documented prior to surgery: surgical approach; fracture characteristics; reduction sequence; temporary stabilization methods; and number, positioning, and dimensions of internal fixation. In addition, the necessity of bone grafting was evaluated. The entire computer-assisted preoperative planning process was converted into an electronic report in PDF format (Supplementary eFig. S2, Supplemental Digital Content, available at: http://links.lww.com/JS9/F218) and sent to each trauma center via email (Fig. 1C). The process of preoperative planning followed the established surgical guideline of the “AO Principles of Fracture Management”^[9]^. Any questions regarding the content of the preoperative planning report were discussed and resolved via remote video conferencing. The electronic report for the computer-assisted preoperative planning was jointly completed by three experienced orthopedic surgeons. All surgical procedures were conducted according to standard methods and a predetermined planning protocol (Fig. 1D). Senior orthopedic surgeons performed the operations. Each surgeon had at least 9 years of experience in performing orthopedic surgery at the outset of this study.

### Outcomes

The primary outcome was the incidence of in-hospital postoperative surgical complication including surgical site infection, urinary tract infection, pneumonia, sepsis, thromboembolic events (deep venous thrombosis or pulmonary embolism), stroke, and myocardial infarction. Secondary outcomes were all-cause in-hospital mortality, duration of surgery measured as the total time elapsed in minutes from incision to the end of suturing, estimated blood loss measured in milliliters from the beginning to the end of the operation, and length of stay considered as continuous variables in days. Other secondary outcomes were the postoperative functional outcomes of the limbs at 12 months after surgery, including the Constant–Murley score (scale: 0–100 points, higher scores indicating better function) for proximal humeral fractures^[19]^, the Mayo Elbow Performance Score (scale: 0–100 points, higher scores representing better function) for distal humeral fractures^[20]^, the Gartland–Werley score [scale: 0–24 points (24 for complete disability; 0 for normal function)] for distal radius fractures^[21]^, the Harris score (scale: 0–100 points, higher scores representing better function) for intertrochanteric fractures^[22]^, the Hospital for Special Surgery (HSS) score [scale: 0–100 points (0 for complete disability; 100 for normal function)] for distal femoral fractures and tibial plateau fractures^[23]^, and the American Orthopedic Foot & Ankle Society (AOFAS) Ankle–Hindfoot Scale (scale: 0–100 points, higher scores indicating better function) for distal tibial fractures and trimalleolar fractures^[24]^. Routine follow-ups were performed at the outpatient clinic at 1, 3, and 12 months after surgery. For patients who were unable to attend in person, follow-up was conducted via text messages or telephone calls.

### Statistical analysis

Quantitative variables were expressed as means with standard deviations (SDs) or medians with interquartile ranges (IQRs), and categorical variables were presented as frequencies and percentages.

To reduce selection bias and potential confounding in baseline characteristics between the conventional method and computer-assisted preoperative planning via telemedicine, propensity score (PS) matching was performed^[25]^. The PSs were calculated using the predicted probability of the PS model after adjusting for potential confounders identified by the directed acyclic graph (Supplemental Digital Content, eFig. S3, available at: http://links.lww.com/JS9/F218)^[26]^, including age, sex, the American Society of Anesthesiologists (ASA) level^[12]^, comorbidity, medication use, smoking status, alcohol use, type of fracture, type of internal fixation, surgical type, timing of surgery, type of anesthesia, surgeon experience, and year of operation. A 1:1 nearest-neighbor matching approach was employed to match patients based on the logit-PS, using a caliper width of 0.25 times the SD of logit-PS^[25]^. The covariate balance between groups was assessed using the standardized mean difference (SMD). An absolute SMD value of <10% was considered acceptable^[27]^. The primary and secondary outcomes of the two groups were compared. The Cox proportional hazards regression analysis was used to compare in-hospital complications and mortality between patients treated with conventional methods and those treated with computer-assisted plans in the PS-matched cohort. Hazard ratios (HRs) were calculated, and robust sandwich estimators were applied to account for clustering within matched sets^[28]^. The proportional hazard assumption was examined using the Schoenfeld residuals test^[29]^. The cumulative incidence of in-hospital complications and survival were estimated using the Kaplan–Meier method. For the analysis of operation duration, blood loss, length of stay, and functional outcomes in the PS-matched cohort, either the independent-sample *t*-test or the Mann–Whitney *U* test was used, depending on the data distribution.

Prespecified subgroup analyses for the primary outcome (in-hospital complications) were performed, stratified by age (18–64 or ≥65 years), sex, ASA status (1, 2, or ≥3), fracture type (proximal humerus, distal humerus, distal radius, proximal femur, distal femur, proximal tibia, distal tibia, and trimalleolar), surgeon experience (9–11, 12–14, or 15–20 years), and year of operation (2010–2012, 2013–2015, or 2016–2019). Four sensitivity analyses were conducted to evaluate the robustness of the findings. First, a Cox proportional hazards regression analysis was conducted to evaluate the association between preoperative planning and primary outcomes in the entire and unadjusted cohorts. Second, we repeated the primary analysis across the entire cohort, including the PS as an additional covariate. Third, the primary analysis of the entire cohort was repeated using the stabilized inverse probability of treatment weighting^[30]^. Finally, the *E*-value was calculated to evaluate the potential effect of unmeasured and residual confounders on the primary outcome (Supplemental Digital Content, eMethod Two and eTable S1, available at: http://links.lww.com/JS9/F218)^[31]^.

Secondary outcomes were considered exploratory due to the potential for Type I errors from multiple comparisons. A two-sided overall *P* value of less than 0.05 was considered statistically significant. All statistical analyses were performed using SAS software (version 9.4; SAS Institute Inc.). An independent statistician blinded to the group assignment conducted all the analyses.

## Results

### Study population

**Figure 2.**
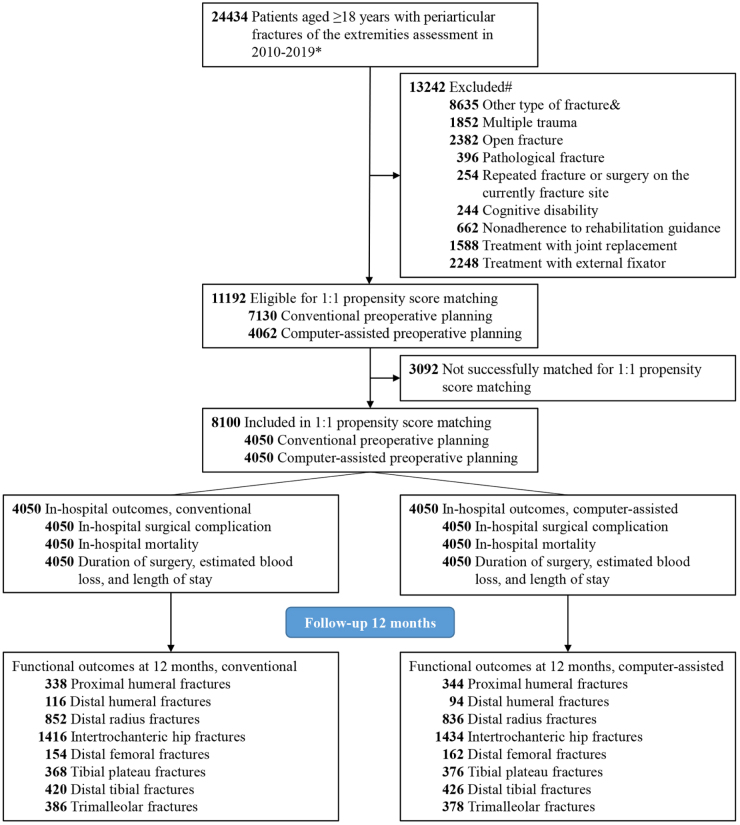
Flow diagram of eligible patients.

In total, 24 434 patients with periarticular fractures of the extremities were identified. Of those, 13 242 patients (54.2%) were excluded based on one or more of the following criteria: other types of fractures (8635 patients), multiple trauma (1852 patients), open fractures (2382 patients), pathological fractures (396 patients), repeated fractures or surgery at the current fracture site (254 patients), cognitive disabilities (244 patients), failure to follow the postoperative rehabilitation guidance (662 patients), treatment with joint replacement (1588 patients) or treatment with external fixator (2248 patients). The final study cohort consisted of 11 192 patients, of which 7130 patients (63.7%) received the conventional method and 4062 patients (36.3%) received computer-assisted preoperative planning via telemedicine (Fig. 2).

* Periarticular fractures of the extremities included proximal humeral fractures, distal humeral fractures, distal radius fractures, intertrochanteric hip fractures, distal femoral fractures, tibial plateau fractures, distal tibial fractures, and trimalleolar fractures.

# Each patient may be included in more than one exclusion group.

& Other type of fracture included AO/OTA (Orthopaedic Trauma Associated) 11-A or 11-B fractures of the proximal humerus, AO/OTA 13-A or 13-B fractures of the distal humerus, AO/OTA 2R3-A or 2R3-B fractures of the distal radius, AO/OTA 31-B or 31-C fractures of the distal femur, AO/OTA 41-A or 41-B fractures of the tibial plateau, AO/OTA 43-A or 43-B fractures of the distal tibia, and AO/OTA 44-A or 44-C fractures of the malleolar segment.

### Patient characteristics

Among 11 192 unadjusted patients, the mean (SD) age was 60.2 (12.3) years, and 7052 patients (63.0%) were female. The variables that differed between the patients who underwent conventional and computer-assisted preoperative planning included dysrhythmia, fracture type, internal fixation type, and surgical type (Table 1). PS matching yielded 4050 patient pairs [mean (SD) age: 60.8 (12.8) years; 5159 patients (63.7%) were female]. After matching, the SMDs for all variables were less than 10%, indicating a good balance between the two groups (Table 1; Supplemental Digital Content, eFig. S4, available at: http://links.lww.com/JS9/F218). The median follow-up time was 14 months (IQR, 12–17) [median (IQR): 14 months^[13–17]^ in the conventional group and 14 months^[12–17]^ in the computer-assisted group].

**Table 1:** Patient characteristics before and after propensity score matching^a^

	Before propensity score matching			After propensity score matching		
Characteristic	Conventional (n = 7130)	Computer-assisted (n = 4062)	SMD,^b^ %	Conventional (n = 4050)	Computer-assisted (n = 4050)	SMD,^b^ %
Age, mean (SD), years	59.9 (11.9)	60.9 (13.0)	8.2	60.8 (12.6)	60.8 (13.0)	−0.3
Female	4477 (62.8)	2575 (63.4)	1.1	2596 (64.1)	2563 (63.3)	−1.7
ASA score^c^						
1	1955 (27.4)	990 (24.4)	−7.0	988 (24.4)	986 (24.3)	−0.1
2	3609 (50.6)	2148 (52.9)	4.5	2122 (52.4)	2144 (52.9)	1.1
≥3	1566 (22.0)	924 (22.7)	1.9	940 (23.2)	920 (22.7)	−1.2
Patient comorbidities						
Hypertension	1622 (22.7)	1058 (26.0)	7.7	1048 (25.9)	1046 (25.8)	−0.1
Diabetes mellitus	716 (10.0)	512 (12.6)	8.1	496 (12.2)	504 (12.4)	0.6
Peripheral vascular disease	300 (4.2)	182 (4.5)	1.3	178 (4.4)	178 (4.4)	0
Chronic kidney disease	138 (1.9)	66 (1.6)	−2.3	70 (1.7)	66 (1.6)	−0.7
COPD	236 (3.3)	150 (3.7)	2.1	160 (4.0)	148 (3.7)	−1.6
Dysrhythmia	496 (7.0)	396 (9.7)	10.1	354 (8.7)	384 (9.5)	2.7
Heart failure	272 (3.8)	110 (2.7)	−6.2	102 (2.5)	110 (2.7)	1.1
Valvular disease	314 (4.4)	198 (4.9)	2.2	176 (4.3)	196 (4.8)	2.3
Liver disease	148 (2.1)	110 (2.7)	4.1	108 (2.7)	110 (2.7)	0.3
Medication use						
Aspirin and/or clopidogrel use	720 (10.1)	460 (11.3)	4.0	446 (11.0)	456 (11.3)	0.8
Corticosteroid use	522 (7.3)	300 (7.4)	0.3	298 (7.4)	298 (7.4)	0
Current smoker	2737 (38.4)	1492 (36.7)	−3.4	1506 (37.2)	1486 (36.7)	−1.0
Alcoholism	2137 (30.0)	1204 (29.6)	−0.7	1202 (29.7)	1204 (29.7)	0.1
Fracture type						
Humerus, proximal	871 (12.2)	344 (8.5)	−12.3	338 (8.3)	344 (8.5)	0.5
Humerus, distal	162 (2.3)	94 (2.3)	0.3	116 (2.9)	94 (2.3)	−3.4
Radius, distal	1670 (23.4)	836 (20.6)	−6.8	852 (21.0)	836 (20.6)	−1.0
Femur, intertrochanteric	2121 (29.7)	1446 (35.6)	12.5	1416 (35.0)	1434 (35.4)	0.9
Femur, distal	202 (2.8)	162 (4.0)	6.4	154 (3.8)	162 (4.0)	1.0
Tibia, proximal	672 (9.4)	376 (9.3)	−0.6	368 (9.1)	376 (9.3)	0.7
Tibia, distal	726 (10.2)	426 (10.5)	1.0	420 (10.4)	426 (10.5)	0.5
Malleoli	706 (9.9)	378 (9.3)	−2.0	386 (9.5)	378 (9.3)	−0.7
Internal fixation type						
Plate and screw	5019 (70.4)	2638 (64.9)	−11.7	2680 (66.2)	2636 (65.1)	−2.3
Intramedullary nail	2111 (29.6)	1424 (35.1)	11.7	1370 (33.8)	1414 (34.9)	2.3
Surgical type						
Open reduction and internal fixation	5201 (72.9)	2776 (68.3)	−10.1	2796 (69.0)	2774 (68.5)	−1.2
Closed reduction and internal fixation	1929 (27.1)	1286 (31.7)	10.1	1254 (31.0)	1276 (31.5)	1.2
Timing of surgery after injury, h						
≤48	1941 (27.2)	1220 (30.0)	6.2	1224 (30.2)	1214 (30.0)	−0.5
>48	5189 (72.8)	2842 (70.0)	−6.2	2826 (69.8)	2836 (70.0)	0.5
Type of anesthesia						
General	6166 (86.5)	3446 (84.8)	−4.7	3416 (84.3)	3436 (84.8)	1.4
Regional	964 (13.5)	616 (15.2)	4.7	634 (15.7)	614 (15.2)	−1.4
Experience of surgeon,^d^ years						
9–11	2035 (28.5)	1272 (31.3)	6.0	1260 (31.1)	1264 (31.2)	0.2
12–14	2375 (33.3)	1304 (32.1)	−2.6	1282 (31.7)	1302 (32.1)	1.1
15–20	2719 (38.1)	1486 (36.6)	−3.2	1508 (37.2)	1484 (36.6)	−1.2
Year of operation						
2010–2012	2122 (29.8)	1152 (28.4)	−3.1	1148 (28.3)	1150 (28.4)	0.1
2013–2015	2357 (33.1)	1372 (33.8)	1.5	1356 (33.5)	1366 (33.7)	0.5
2016–2019	2651 (37.2)	1538 (37.9)	1.4	1546 (38.2)	1534 (37.9)	−0.6

SMD, standardized mean difference; SD, standard deviation; ASA, American Society of Anesthesiologists; COPD, chronic obstructive pulmonary disease.

^a^ Data are presented as the number (percentage) of patients unless otherwise indicated. Percentages may not total 100 because of rounding.

^b^ Standardized mean difference is the absolute difference in means or percentage divided by an evenly weighted pooled SD, or the difference between groups in the number of SDs, expressed as percentage.

^c^ Range, 1–6; higher level indicates greater risk during anesthesia. ASA 1 indicating a healthy patient with no disease, ASA 2 indicating a patient with mild systemic disease, ASA 3 indicating a patient with severe systemic disease, ASA 4 indicating a patient with severe systemic disease that is life-threatening, ASA 5 indicating a patient who is not expected to survive without surgery, and ASA 6 indicating a patient in whom brain death has occurred.

^d^ Data represent the years of clinical experience of the surgeon at the time of surgery.

### Primary analysis

**Figure 3.**
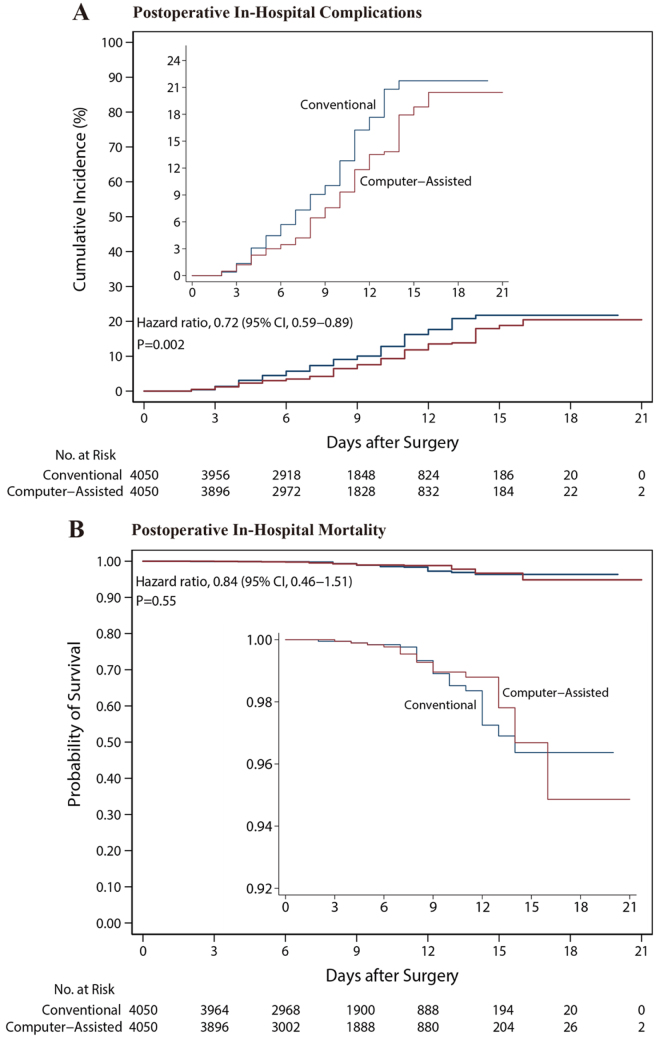
Cumulative risks of in-hospital complications (A) and Kaplan–Meier survival curves (B) in propensity score-matched patients with conventional or computer-assisted preoperative planning via telemedicine.

After matching, patients who underwent computer-assisted preoperative planning via telemedicine had a lower rate of in-hospital complications [7.9% (318/4050)] compared with those with conventional planning [10.9% (442/4050)] (HR, 0.72; 95% CI: 0.59–0.89; *P* = 0.002) (Table 2 and Fig. 3A).

The insets show the same data on an expanded *y*-axis.

**Table 2:** Primary and secondary outcomes in propensity-score matched patients^a^

Outcome	Conventional (n = 4050)^b^		Computer-assisted (n = 4050)^b^		Mean group-between difference (95% CI)	Hazard ratio (95% CI)	*P* value
	No. of patient		No. of patient		Percentage points	Percentage points	
Primary outcome							
In-hospital complications^c^	4050	442 (10.9)	4050	318 (7.9)	-	0.72 (0.59–0.89)	0.002
Surgical site infection	4050	156 (3.9)	4050	114 (2.8)	-	-	-
Urinary tract infection	4050	130 (3.2)	4050	80 (2.0)	-	-	-
Pneumonia	4050	138 (3.4)	4050	74 (1.8)	-	-	-
Sepsis	4050	84 (2.1)	4050	64 (1.6)	-	-	-
Thromboembolic event	4050	104 (2.6)	4050	76 (1.9)	-	-	-
Stroke	4050	58 (1.4)	4050	34 (0.8)	-	-	-
Myocardial infarction	4050	38 (0.9)	4050	20 (0.5)	-	-	-
Secondary outcome							
In-hospital mortality	4050	48 (1.2)	4050	40 (1.0)	-	0.84 (0.46–1.51)	0.55
Duration of surgery, min	4050	100.0 (92.0–128.0)	4050	69.0 (60.0–93.0)	−33.8 (−35.4 to −32.2)	-	<0.001
Estimated blood loss, mL	4050	150.0 (100.0–170.0)	4050	100.0 (80.0–110.0)	−51.6 (−54.0 to −49.3)	-	<0.001
Length of stay, days	4050	8.0 (5.0–11.0)	4050	8.0 (5.0–11.0)	−0.07 (−0.30 to 0.15)	-	0.53
Constant–Murley score for proximal humeral fractures^d^	338	65.7 (4.0)	344	74.5 (3.9)	8.9 (8.0–9.7)	-	<0.001
Mayo score for distal humeral fractures^e^	116	76.3 (4.8)	94	85.7 (6.6)	9.4 (7.1–11.7)	-	<0.001
Gartland–Werley score for distal radius fractures^f^	852	6.7 (2.0)	836	2.6 (1.1)	−4.1 (−4.3 to −3.9)	-	<0.001
Harris score for intertrochanteric fractures^g^	1416	65.2 (8.1)	1436	65.6 (7.1)	0.38 (−0.41 to 1.17)	-	0.34
HSS score for distal femoral fractures^h^	154	74.1 (7.4)	162	84.4 (8.1)	10.3 (7.9–12.7)	-	<0.001
HSS score for tibial plateau fractures^h^	368	80.6 (5.4)	376	87.7 (5.9)	7.1 (6.0–8.3)	-	<0.001
AOFAS score for distal tibial fractures^i^	420	75.9 (7.0)	426	86.0 (7.6)	10.1 (8.7–11.5)	-	<0.001
AOFAS score for trimalleolar fractures^i^	386	78.1 (6.8)	378	92.0 (6.7)	13.9 (12.6–15.3)	-	<0.001

CI, confidence interval; HSS, Hospital for Special Surgery; AOFAS, American Orthopaedic Foot & Ankle Society.

^a^ For in-hospital complications and mortality, hazard ratio is presented; for continuous outcomes, the mean group-between difference is presented. Hazard ratios and *P* values for in-hospital complications and mortality are calculated with a Cox proportional hazards model in the propensity score-matched cohort; for continuous outcomes, *P* values are based on the independent-sample *t*-test or the Mann–Whitney *U* test in the propensity score-matched cohort.

^b^ Data for in-hospital complications and mortality are expressed as number (percentage) of patients; data for continuous outcomes are expressed as mean (standard deviation) or median (interquartile range) of patients.

^c^ Some patients may experience more than one in-hospital complications.

^d^ Range, 0–100, with higher scores indicating better function.

^e^ Range, 0–100, with higher scores indicating better function.

^f^ Range, 0–24, 0 representing normal function and 24 representing complete disability.

^g^ Range, 0–100, with higher scores indicating better function.

^h^ Range, 0–100, with higher scores indicating better function.

^i^ Range, 0–100, with higher scores indicating better function.

### Secondary analysis

**Figure 4.**
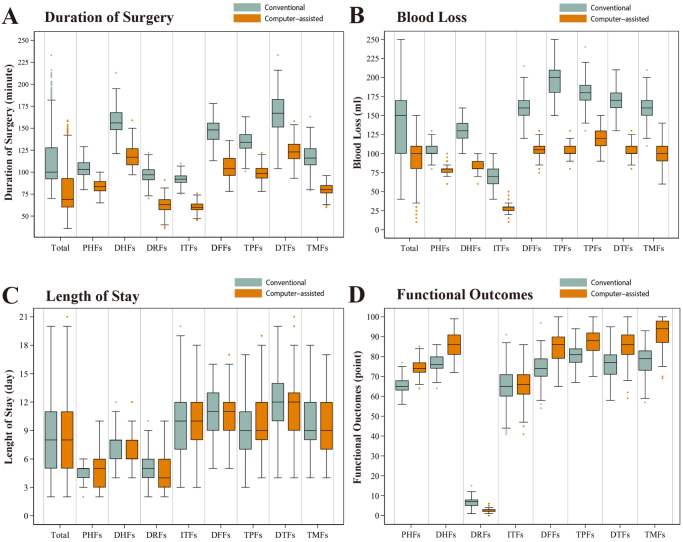
Duration of surgery (A), estimated blood loss (B), length of stay (C), and functional outcomes (D) in propensity score-matched patients.

Among the 8100 matched patients, there was no statistically significant difference between those receiving conventional preoperative planning and those with computer-assisted planning in in-hospital mortality [conventional: 1.2% (48/4050 patients) vs. computer-assisted: 1.0% (40/4050 patients); HR: 0.84 (0.46–1.51); *P* = 0.55] (Table 2 and Fig. 3B) and length of stay [conventional: 8.0 days (IQR, 5.0–11.0) vs. computer-assisted: 8.0 days (IQR: 5.0–11.0); mean difference: −0.07 days (95% CI: −0.30 to 0.15); *P* = 0.53] (Table 2 and Fig. 4C). Patients in the computer-assisted group were associated with a shorter duration of surgery (100.0 minutes (IQR: 92.0–128.0) vs. 69.0 minutes (IQR, 60.0–93.0); mean difference: −33.8 minutes (95% CI: −35.4 to −32.2); *P* < 0.001] (Table 2 and Fig. 4A) and less estimated blood loss [150.0 minutes (IQR, 100.0–170.0) vs. 100.0 minutes (IQR: 80.0–110.0); mean difference: 51.6 minutes (95% CI: −54.0 to −49.3); *P* < 0.001] compared with those in the conventional group (Table 2 and Fig. 4B).

The box represents the median and interquartile range, the whiskers represent 1.5 times the interquartile range beyond the 25th and 75th percentiles, and the dots represent the more extreme values.

Abbreviations: PHFs, proximal humeral fractures; DHFs, distal humeral fractures; DRFs, distal radius fractures; ITFs, intertrochanteric fractures; DFFs, distal femoral fractures; TPFs, tibial plateau fractures; DTFs, distal tibial fractures; TMFs, trimalleolar fractures.

After matching, patients in the computer-assisted group were associated with improved functional outcomes at 12 months compared with those in the conventional group, including the Constant score for proximal humeral fractures [65.7 points for 338 patients in the conventional group vs. 74.5 points for 344 patients in the computer-assisted group; mean difference, 8.9 points (95% CI: 8.0–9.7); *P* < 0.001], the Mayo score for distal humeral fractures [76.3 points for 116 patients in the conventional group vs. 85.7 points for 94 patients in the computer-assisted group; mean difference: 9.4 points (95% CI: 7.1–11.7); *P* < 0.001], the Gartland–Werley score for distal radius fractures [6.7 points for 852 patients in the conventional group vs. 2.6 points for 1436 patients in the computer-assisted group; mean difference: −4.1 points (95% CI: −4.3 to −3.9); *P* < 0.001], the HSS score for distal femoral fractures [74.1 points for 154 patients in the conventional group vs. 84.4 points for 162 patients in the computer-assisted group; mean difference: 10.3 points (95% CI: 7.9–12.7); *P* < 0.001], the HSS score for tibial plateau fractures [80.6 points for 368 patients in the conventional group vs. 87.7 points for 376 patients in the computer-assisted group; mean difference: 7.1 points (95% CI: 6.0–8.3); *P* < 0.001], the AOFAS score for distal tibial fractures [75.9 points for 420 patients in the conventional group vs. 86.0 points for 426 patients in the computer-assisted group; mean difference, 10.1 points (95% CI: 8.7–11.5); *P* < 0.001], and the AOFAS score for trimalleolar fractures [78.1 points for 386 patients in the conventional group vs. 92.0 points for 378 patients in the computer-assisted group; mean difference, 13.9 points (95% CI: 12.6–15.3); *P* < 0.001] (Table 2 and Fig. 4D). However, no significant difference in Harris scores was observed for patients with intertrochanteric fractures [65.2 points for 1416 patients in the conventional group vs. 65.6 points for 162 patients in the computer-assisted group; mean difference, 0.38 points (95% CI: −0.41 to 1.17); *P* = 0.34] (Table 2 and Fig. 4D).

### Subgroup and sensitivity analyses

**Figure 5.**
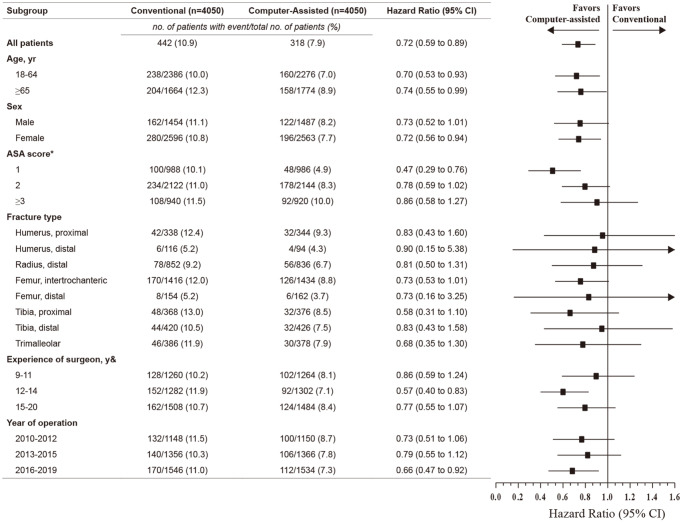
Prespecified subgroup analysis of in-hospital complications in propensity-score matched patients.

The subgroup analyses of the primary outcomes were consistent with the main findings (Fig. 5). Results of the sensitivity analysis for in-hospital complications in the crude, unadjusted cohort were consistent with the results of the primary analysis [11.0% (786/7130 patients) in the conventional group vs. 7.8% (318/4062 patients) in the computer-assisted group; HR: 0.66 (95% CI: 0.55–0.79); *P* < 0.001]. Additional PS-adjusted analysis yielded results similar to the main findings [HR, 0.65 (95% CI: 0.54–0.78); *P* < 0.001]. Results from stabilized inverse probability of treatment weighting aligned with the primary analysis [HR: 0.66 (95% CI: 0.55–0.79); *P* < 0.001]. The *E*-value for the point estimate was 2.12, indicating that the observed HR of 0.72 could be explained by an unmeasured confounder.

& Data represent the years of clinical experience of the surgeon at the time of surgery.

## Discussion

This cohort study involving patients with complex periarticular fractures of the extremities showed that those who received computer-assisted preoperative planning via digital telemedicine had a significantly lower incidence of in-hospital complications (surgical site infection, urinary tract infection, pneumonia, sepsis, thromboembolic events, stroke, and myocardial infarction) than those who underwent conventional methods; however, no significant difference was observed in in-hospital mortality. Patients who underwent computer-assisted preoperative planning had shorter surgery duration, less estimated blood loss, and better functional outcomes at 12 months.

**Figure 6.**
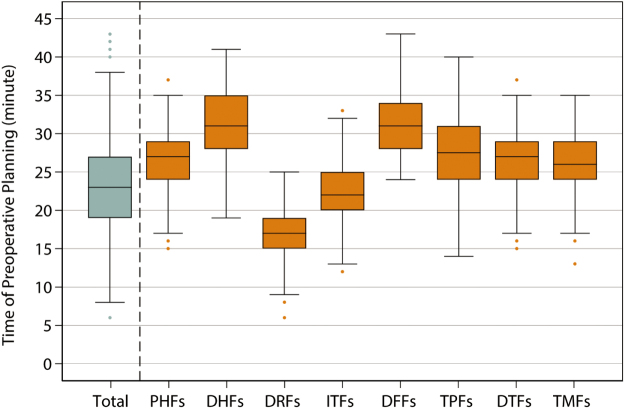
Total time of preoperative planning based on computer-assisted virtual technology (light gray box) and independent time of preoperative planning of each part of fracture (orange box). The box represents the median and interquartile range, the whiskers represent 1.5 times the interquartile range beyond the 25th and 75th percentiles, and the dots represent the more extreme values.

Abbreviations: PHFs, proximal humeral fractures; DHFs, distal humeral fractures; DRFs, distal radius fractures; ITFs, intertrochanteric fractures; DFFs, distal femoral fractures; TPFs, tibial plateau fractures; DTFs, distal tibial fractures; TMFs, trimalleolar fractures.

The application of digital telemedicine in orthopedics is predominantly observed in postoperative rehabilitation guidance and nursing care following orthopedic-related surgeries^[32–34]^. However, this study is the first to integrate a digital telemedicine system into the entire process of computer-assisted preoperative fracture planning, achieving collaborative operations across five trauma centers. This model overcomes the geographical limitations of traditional CT-based preoperative planning and solves the geographical accessibility problem of traditional models^[10,12,13]^. This innovative workflow has revolutionary significance for orthopedic centers in resource-limited areas. Previous studies on computer-assisted orthopedic preoperative planning have mainly been conducted at a single center or on a small scale^[10–12]^. Although the advantages of computer-assisted orthopedic preoperative planning over traditional preoperative planning have been confirmed^[10–12,35]^, no studies have confirmed whether this model can be applied remotely to address the problem of accessibility to high-quality preoperative planning resources. This study validated the feasibility of efficiently distributing and executing high-quality preoperative planning via remote collaboration, with computer-assisted preoperative planning taking an average of approximately 30 minutes (Fig. 6).

This study has clear advantages over previous studies in terms of the fracture type (eight complex periarticular fractures in different locations) and sample size (*n* = 11 192)^[10–13]^. Previous studies have mostly focused on a single fracture type, and the number of cases was small^[10,11,13]^. Although the diversity of fracture types increases heterogeneity, this precisely reflects the actual clinical challenges. This was also one of the purposes of the study, that is, to evaluate the generalizability and overall benefits of this remote planning model for a variety of complex fracture types. The results demonstrated that the patients who underwent computer-assisted preoperative planning via telemedicine had a lower risk of in-hospital complications and better postoperative functional outcomes than those who underwent conventional planning alone. The advantages of computer-assisted preoperative planning stem from several key factors. First, the electronic reports it generates enhance surgeons’ ability to precisely delineate fracture characteristics. This facilitates the detailed assessment of fracture line morphology, identification of fragment number and location, evaluation of articular surface depression and comminution, verification of potential bone defects, and determination of bone graft requirements. Second, this technology provides surgeons with highly accurate preoperative insights into the required internal fixation, including implant type, number, size, and optimal positioning. This information enables a rapid, precise intraoperative fracture reduction and guides the optimal selection and placement of fixation devices. Consequently, this approach enhances the success rate of reduction and fixation while minimizing unnecessary repetitive intraoperative maneuvers. As evidenced by this study’s findings, these efficiencies translate directly into reduced intraoperative reduction time, fewer fluoroscopic exposures, shortened overall operative duration, and diminished blood loss. Third, the accurate preoperative assessment of fracture morphology, coupled with a thorough understanding of the planned fixation strategy, mitigates iatrogenic damage to surrounding soft tissues and the peri-fracture vascular supply. Furthermore, the aforementioned reductions in surgical time and blood loss contribute additively to minimizing overall surgical trauma.

No significant differences in in-hospital mortality were observed between the two groups. This may be attributed to the well-established comprehensive perioperative management as well as the lack of differences in age, sex, and comorbidities between the groups. However, this was a comprehensive result of multiple fractures, and the outcomes of individual fractures may vary. However, further investigation is required. There was no significant difference in the length of hospital stay between the two groups, as the patients were required to wait until there was no obvious exudation or swelling at the surgical incision before discharge.

This study has some limitations. First, considering the observational design, there was a potential for unmeasured confounding factors and bias. Therefore, PS matching, subgroup analyses, and sensitivity analyses were performed to reduce confounding bias. Second, some potential confounding factors that may have influenced the results could not be identified. Therefore, the *E*-value was calculated to assess the effect of residual confounders on the results, and it was observed that residual confounders did not alter the conclusions of the main analysis. Despite these procedures, further investigations are needed in larger randomized prospective studies. Third, postdischarge complications may have arisen; however, the data sources used in this analysis did not capture information on complications or mortality after discharge. Finally, although the computer-assisted preoperative planning process in this study did not require direct execution by clinicians, some surgeons may consider it time-consuming and cumbersome, making it impractical in clinical practice. However, this was not the case in the present study. In this study, the average duration of virtual planning was approximately half an hour, and all procedures were completed within a single system, demonstrating the efficiency and convenience of the computer-assisted preoperative planning system.

## Conclusion

Among patients with complex periarticular fractures of the extremities, computer-assisted preoperative planning via digital telemedicine was associated with a lower incidence of in-hospital complications and improved functional outcomes compared to conventional methods. Additionally, computer-assisted planning reduced surgery duration and estimated blood loss. This study demonstrated that a remote collaboration model of computer-assisted preoperative planning based on digital telemedicine is both feasible and effective, helping to solve the problem of limited access to high-quality preoperative planning resources. These findings support the use of computer-assisted preoperative planning using digital telemedicine in the treatment of systemic bone and joint fractures.

## Data Availability

The data that support the findings of this study are available on reasonable request from the corresponding author. The data are not publicly available due to privacy and ethical restrictions.

## Research registration unique identifying number (UIN)

Name of the registry: research registry. Unique Identifying number or registration ID: researchregistry11122. Hyperlink to your specific registration (must be publicly accessible and will be checked): https://researchregistry.knack.com/researchregistry#home/registrationdetails/67e134afb4d5da0312d9f7aa/.
